# Choice or no choice? The need for better branded public sector condoms in South Africa

**DOI:** 10.4102/sajhivmed.v16i1.353

**Published:** 2015-07-02

**Authors:** John Ashmore, Ruth Henwood

**Affiliations:** 1 Doctors Without Borders/Médecins Sans Frontières, Cape Town, South Africa; 2 Doctors Without Borders/Médecins Sans Frontières, Khayelitsha, South Africa

## Abstract

Condoms are one of the cornerstones to any response to the HIV epidemic. However, targeted marketing strategies that make condoms more attractive to people at high risk of infection are often overlooked. The South African National Department of Health has recently purchased more attractive condoms to distribute in higher-education settings free of charge, targeting at-risk youth including young women. The authors applaud this move but note the importance of expanding better branded condoms to young people elsewhere – for example, via youth clinics and in high schools. Exploratory, routine data from Médecins Sans Frontières in Khayelitsha are presented, showing the popularity of alternatives to the government's ‘Choice’ brand.

## Introduction

### Background

Condom distribution, promotion and social marketing represent a highly cost-effective HIV prevention strategy, given the low cost of condoms and their strong prevention efficacy. Self-reported condom usage is around 80% effective at preventing HIV for heterosexual couples, 70% effective for men who have sex with men (MSM), and much higher under laboratory conditions.^1^ Condoms are advisable even for seroconcordant couples, to help prevent other sexually transmitted infections and unwanted pregnancy.^2^ Lubricant is also advised for use with condoms. According to the World Health Organization, water-based lubricants are associated with either a decrease or no change in the rate of condom slippage or breakage. Female condoms are also considered safe.^3^

The latest Human Sciences Research Council behaviour survey,^4^ however, found that self-reported condom use at time of last sex significantly declined in South Africa, from 45.1% (95% confidence interval [CI] 43.3–47.0) of adults in 2008, to 36.2% (95% CI 34.5–37.9) in 2012. This decline is in spite of increases in the total number of male condoms distributed by the public sector, over the same period, from 283 000 ^5^ to 501 000.^6^ Female condoms still represent a small fraction of total condoms distributed.

Importantly, greater distribution of condoms does not ensure their increased and proper use, especially if divorced from a well-considered, segmented social marketing campaign. Such a campaign would ideally: (1) research the needs and preferences of high risk and general populations, (2) deliver heterogeneous products branded differently for different target groups and (3) provide public messaging promoting healthy behaviour appealing to the target audience.^7,8^

The South African government's free ‘Choice’ brand condoms, meanwhile, do not target any specific population or form part of any official marketing strategy. The main condom brands available at present are Choice (80% of the market) followed by Population Service International's subsidised ‘Trust’ and ‘Lovers+’^9^ brands.

Partly in response to such issues, Minister of Health Dr Motsoaledi announced in April 2014 that free, colourful and flavoured condoms would be provided in South African tertiary education institutions. The minister noted that this was to combat ‘condom fatigue’, referring to a lack of enthusiasm amongst young people for Choice.^10^ Since this announcement, the National Department of Health awarded a tender for 50 million rebranded condoms for universities and further education and training colleges and has begun distributing them. However, by the age of 21, 50% of young people are not employed or enrolled in any form of education (20% at age 18) and thus do not benefit equally.^11^ School age adolescents, particularly young women, are also vulnerable to HIV infection. A recent development is that the Department of Basic Education has drafted a policy to provide condoms in schools but faces backlash from school boards and religious groups who see this as encouraging sex,^12,13^ rather than safe sex, despite a lack of evidence supporting this claim.^14^

The present editorial calls for the further expansion of attractively branded and marketed condoms via the public sector, especially to meet the needs of young, marginalised people, many of whom are not enrolled in tertiary education. Exploratory, routine data are presented from Médecins Sans Frontières (MSF) in Khayelitsha that adds weight to these arguments.

## Condoms in Khayelitsha

**FIGURE 1:**
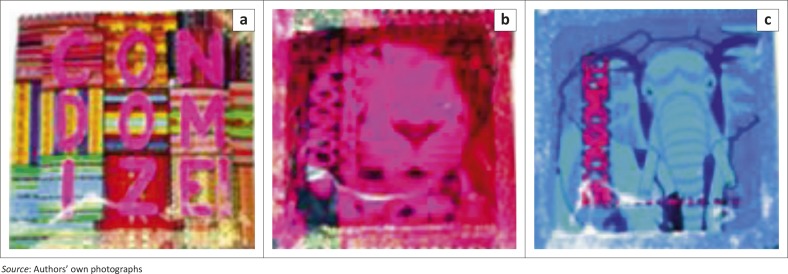
Photo's of (a) Condomize regular, (b) flavoured and (c) extra-large.

Khayelitsha township has one of the most successful condom distribution programmes in the country, with approximately 1 million Choice condoms distributed per month by the Treatment Action Campaign to a population of 391 749.^15^ With permission from the City of Cape Town, MSF conducted a trial on the popularity of alternatives to Choice in a youth clinic in Khayelitsha, using condoms donated by the organization ‘Condomize’. Four condom types (Choice, Condomize regular with bright packaging, Condomize strawberry flavoured, and Condomize extra-large [Figure 1]) were placed in identical glass containers in the reception area of Site C Youth Clinic. Condom containers were refilled under the supervision of MSF's Patient Support team. Placing the condoms in the waiting area of the clinic allowed youth access with minimal interference from health workers. Clients could take as many or as few individually packed condoms as desired.

**FIGURE 2:**
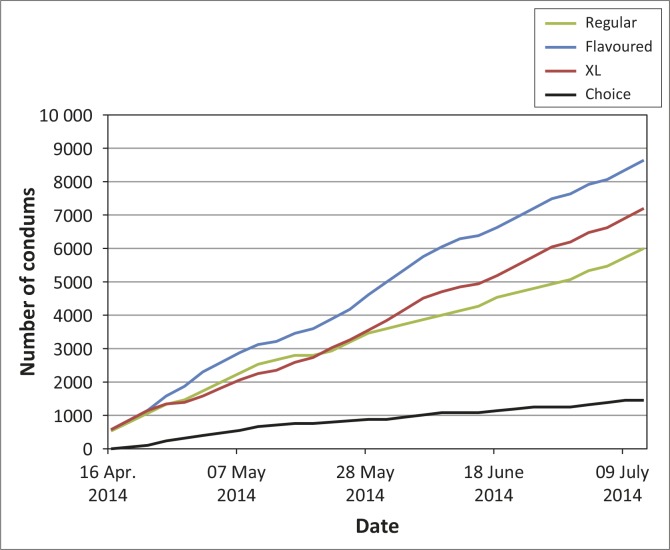
Cumulative total of condoms taken in Site C Youth Clinic: moving average.

As shown in Figure 2, flavoured condoms proved the most popular, followed closely by extra-large condoms. Interestingly, even the regular condoms in bright packaging were far more popular than Choice, although their popularity seemed to decline slightly over time. Only 6% of condoms taken from the youth clinic were Choice condoms.

In discussions with youths about the new condoms at the clinic, the overall feeling was one of satisfaction. They preferred the colourful packaging, smell, fit and feel which some described as ‘lighter’. Further remarks were ‘Choice condoms are too thick and oily’, ‘we experienced pain due to the poor fit’, and ‘the old ones smelled awful and didn’t fit well’. Some young women indicated that their boyfriends would sometimes buy condoms rather than use the freely available Choice condoms. This anecdotal evidence implies higher acceptability of alternative brands to Choice, at least in the short term.

## Recommendations

Although the experience of Khayelitsha may not be generalisable to elsewhere in South Africa, changes to packaging and marketing of government condoms may nevertheless prove popular with South African youth. Repackaged, flavoured and extra-large condoms, in particular, appear to show some promise. The South African government should be praised for their introduction of such condoms to tertiary education institutions but must ensure wider distribution and reconsider their overall marketing strategy. MSF has seen high demand in its projects for alternative condoms, along with family planning, amongst school age adolescents. In terms of introducing condoms in schools and other outlets such as youth clinics, it is time to leave behind moralistic arguments about sex and focus on addressing the public health crisis at hand. The costs of rebranding and marketing condoms should be weighed against the potential benefits of increased popularity and use.

In general, more research is needed to explore condom market dynamics in South Africa and elsewhere, including focusing on differences in design preference by gender, age and HIV risk. It is also important to identify whether alternative designs are preferred in the long term or whether there is a need for regular design changes to maintain novelty. Such research should inform a national condom marketing strategy that maximises the impact of free government condoms which might no longer capture the imagination of their intended users. Young people, but also sex workers, MSM and prisoners, may all require their own strategies, along with a new approach for the general population. Lubricant should also be considered for distribution along with condoms, especially for MSM, while there is also much scope for social marketing messages that promote correct and consistent condom use. It is unknown at present how many people who collect the various brands of condoms will actually use them. This point may be particularly important to consider in a setting where intimate partner violence and the inability of women to negotiate condom access are linked to HIV infection.^16^
